# Supplementary material for: Genetic population structure of the alpine species Rhododendron pseudochrysanthum sensu lato (Ericaceae) inferred from chloroplast and nuclear DNA
Source: BMC Evol Biol. 2011 Apr 19;11:108. doi: 10.1186/1471-2148-11-108 (PMC3096940; doi:10.1186/1471-2148-11-108)
Supplement: Additional file 1 — Table S1. Absolute frequency of chloroplast DNA haplotypes of Rhododendron pseudochrysanthum s.l. Numbers of chloroplast haplotypes distributions from 14 populutions of Rhododendron pseudochrysanthum s.l. Cluster I and II are indicated. N: number of samples. Alishan Mountain Range: AA; Central Mountain Range: CB, CK, CY, CS, CN, CH; Sheishan Mountain Range: SS, SL, SB, SC; Yushan Mountain Range: YT, YY, YP. See Table 1 for the detailed information of populations. [file 1471-2148-11-108-S1.DOC]

Additional file 1: Table S1 - **Absolute frequency of chloroplast DNA haplotypes of *Rhododendron pseudochrysanthum* s.l.** Numbers of chloroplast haplotypes distributions from 14 populutions of *Rhododendron pseudochrysanthum* s.l. Cluster I and II are indicated. N: number of samples. Alishan Mountain Range: AA; Central Mountain Range: CB, CK, CY, CS, CN, CH; Sheishan Mountain Range: SS, SL, SB, SC; Yushan Mountain Range: YT, YY, YP. See Table 1 for the detailed information of populations.

| Cluster | Haplotype | AA | CB | CK | CY | CS | CN | CH | SS | SL | SB | SC | YT | YY | YP |
| --- | --- | --- | --- | --- | --- | --- | --- | --- | --- | --- | --- | --- | --- | --- | --- |
| I | PH01 | 4 |  | 1 | 3 | 4 | 2 | 2 | 2 | 3 | 4 |  |  |  |  |
| I | PH02 |  | 3 | 1 |  | 1 |  |  |  |  |  |  |  |  |  |
| I | PH03 |  | 1 |  |  |  |  |  |  |  |  |  |  |  |  |
| Ia | PH04 |  |  |  |  |  |  |  |  |  |  |  | 3 | 1 | 1 |
| Ia | PH05 |  |  |  |  |  |  |  |  |  |  |  |  |  | 1 |
| Ia | PH06 |  |  |  |  |  |  |  |  |  |  |  |  | 1 | 1 |
| Ia | PH07 |  |  |  |  |  |  |  |  |  |  |  |  |  | 1 |
| Ia | PH08 |  |  |  |  | 1 |  |  |  |  |  |  |  |  |  |
| I | PH09 |  |  |  |  | 1 |  |  |  |  |  |  |  |  |  |
| Ia | PH10 |  |  |  |  |  |  |  |  |  |  |  | 1 |  | 1 |
| Ia | PH11 |  |  |  |  |  |  |  |  |  |  |  | 1 |  |  |
| Ia | PH12 |  |  |  |  |  |  |  |  |  |  |  | 1 |  |  |
| Ia | PH13 |  |  |  |  |  |  |  |  |  |  |  | 1 |  |  |
| Ia | PH14 |  |  |  |  |  |  |  |  |  |  |  | 1 |  |  |
| Ia | PH15 |  |  |  |  |  |  |  |  |  |  |  | 1 |  |  |
| I | PH16 |  |  |  |  |  | 1 |  |  |  |  |  |  |  |  |
| I | PH17 |  |  |  |  |  | 1 |  |  |  |  |  |  |  |  |
| Ia | PH18 |  |  |  | 1 |  |  |  |  |  |  |  |  |  |  |
| Ia | PH19 |  |  | 1 |  |  |  |  |  |  |  |  |  |  |  |
| Ib | PH20 |  |  | 1 |  |  |  |  |  |  |  |  |  |  |  |
| II | PH21 |  |  |  |  |  | 1 |  |  |  |  |  |  |  |  |
| II | PH22 |  |  |  |  |  | 1 |  |  |  |  |  |  |  |  |
| Ia | PH23 |  |  |  |  |  |  |  |  |  |  |  |  | 1 |  |
| Ia | PH24 |  |  |  |  |  |  |  |  |  |  |  |  | 1 |  |
| Ia | PH25 |  |  |  |  |  |  |  |  |  |  |  |  | 1 |  |
| Ib | PH26 |  |  |  |  |  |  |  |  |  |  |  |  | 1 |  |
| I | PH27 |  |  |  |  | 1 |  |  |  |  |  |  |  |  |  |
| I | PH28 |  |  |  |  |  |  |  | 1 |  |  |  |  |  |  |
| I | PH29 |  |  |  |  |  |  |  | 1 |  |  |  |  |  |  |
| I | PH30 |  |  |  |  |  |  | 1 |  |  |  |  |  |  |  |
| I | PH31 |  |  |  |  |  |  | 1 |  |  |  |  |  |  |  |
| I | PH32 |  |  |  |  |  |  |  |  |  | 1 |  |  |  |  |
| I | PH33 |  |  |  |  |  |  |  |  |  | 1 |  |  |  |  |
| I | PH34 |  |  |  |  |  |  |  |  |  |  | 1 |  |  |  |
| I | PH35 |  |  |  |  |  |  |  |  | 1 |  |  |  |  |  |
| I | PH36 |  |  |  |  |  |  |  |  | 1 |  |  |  |  |  |
|  | N | 4 | 4 | 4 | 4 | 8 | 6 | 4 | 4 | 5 | 6 | 1 | 9 | 6 | 5 |
