# Supplementary material for: Genetic population structure of the alpine species Rhododendron pseudochrysanthum sensu lato (Ericaceae) inferred from chloroplast and nuclear DNA
Source: BMC Evol Biol. 2011 Apr 19;11:108. doi: 10.1186/1471-2148-11-108 (PMC3096940; doi:10.1186/1471-2148-11-108)
Supplement: Additional file 2 — Table S2. Absolute frequency of nuclear DNA haplotypes of Rhododendron pseudochrysanthum s.l. Numbers of nuclear haplotypes distributions from 14 populutions of Rhododendron pseudochrysanthum s.l. Cluster A, B and C are indicated. N: number of samples. Alishan Mountain Range: AA; Central Mountain Range: CB, CK, CY, CS, CN, CH; Sheishan Mountain Range: SS, SL, SB, SC; Yushan Mountain Range: YT, YY, YP. See Table 1 for the detailed information of populations. [file 1471-2148-11-108-S2.DOC]

Additional file 2: Table S2 - **Absolute frequency of nuclear DNA haplotypes of *Rhododendron pseudochrysanthum* s.l.** Numbers of nuclear haplotypes distributions from 14 populutions of *Rhododendron pseudochrysanthum* s.l. Cluster A, B and C are indicated. N: number of samples. Alishan Mountain Range: AA; Central Mountain Range: CB, CK, CY, CS, CN, CH; Sheishan Mountain Range: SS, SL, SB, SC; Yushan Mountain Range: YT, YY, YP. See Table 1 for the detailed information of populations.

| Cluster | Haplotype | AA | CB | CK | CY | CS | CN | CH | SS | SL | SB | SC | YT | YY | YP |
| --- | --- | --- | --- | --- | --- | --- | --- | --- | --- | --- | --- | --- | --- | --- | --- |
| A | PR01 | 2 |  |  |  |  |  |  |  |  |  |  |  |  |  |
| A | PR02 | 1 |  |  |  |  |  |  |  |  |  |  |  |  |  |
| A | PR03 | 1 |  |  |  |  |  |  |  |  |  |  |  |  |  |
| A | PR04 |  | 1 |  |  |  |  |  |  |  |  |  |  |  |  |
| A | PR05 |  | 3 |  |  |  |  |  | 3 |  |  |  |  |  |  |
| A | PR06 |  |  | 2 | 1 | 1 | 1 | 2 |  | 2 | 2 |  | 3 |  | 2 |
| A | PR07 |  |  |  |  | 1 |  |  |  |  |  |  |  |  |  |
| A | PR08 |  |  |  |  |  | 1 |  |  |  |  |  |  |  |  |
| A | PR09 |  |  |  |  | 1 |  |  |  |  |  |  |  |  |  |
| A | PR10 |  |  | 1 |  | 3 | 1 |  |  |  |  |  | 2 | 2 |  |
| A | PR11 |  |  |  |  | 1 |  |  |  |  |  |  |  |  |  |
| C | PR12 |  |  |  |  | 1 |  |  |  |  |  |  |  |  |  |
| B | PR13 |  |  |  |  |  |  |  |  |  |  |  |  |  | 1 |
| A | PR14 |  |  |  |  |  |  |  |  |  |  |  |  |  | 1 |
| A | PR15 |  |  |  |  |  |  |  |  |  |  |  |  |  | 1 |
| B | PR16 |  |  |  |  |  |  |  |  |  |  |  | 2 |  |  |
| B | PR17 |  |  |  |  |  |  |  |  |  |  |  | 2 |  |  |
| A | PR18 |  |  |  |  |  |  | 1 |  |  |  |  |  |  |  |
| A | PR19 |  |  |  |  |  |  | 1 |  |  |  |  |  |  |  |
| A | PR20 |  |  |  | 1 |  |  |  |  |  |  |  |  |  |  |
| A | PR21 |  |  |  | 1 |  |  |  |  |  |  |  |  |  |  |
| A | PR22 |  |  |  | 1 |  |  |  |  |  |  |  |  |  |  |
| A | PR23 |  |  | 1 |  |  |  |  |  |  |  |  |  |  |  |
| A | PR24 |  |  |  |  |  | 1 |  |  |  |  |  |  |  |  |
| A | PR25 |  |  |  |  |  | 1 |  |  |  |  |  |  |  |  |
| A | PR26 |  |  |  |  |  | 1 |  |  |  |  |  |  |  |  |
| A | PR27 |  |  |  |  |  |  |  | 1 |  |  |  |  |  |  |
| B | PR28 |  |  |  |  |  |  |  |  |  |  |  |  | 2 |  |
| A | PR29 |  |  |  |  |  |  |  |  |  |  |  |  | 2 |  |
| A | PR30 |  |  |  |  |  |  |  |  |  | 2 |  |  |  |  |
| A | PR31 |  |  |  |  |  |  |  |  |  | 2 |  |  |  |  |
| A | PR32 |  |  |  |  |  |  |  |  |  |  | 1 |  |  |  |
| B | PR33 |  |  |  |  |  |  |  |  | 1 |  |  |  |  |  |
| C | PR34 |  |  |  |  |  |  |  |  | 1 |  |  |  |  |  |
| C | PR35 |  |  |  |  |  |  |  |  | 1 |  |  |  |  |  |
|  | N | 4 | 4 | 4 | 4 | 8 | 6 | 4 | 4 | 5 | 6 | 1 | 9 | 6 | 5 |
